# An ultralong-acting tenofovir ProTide nanoformulation achieves monthslong HBV suppression

**DOI:** 10.1126/sciadv.ade9582

**Published:** 2022-12-23

**Authors:** Srijanee Das, Weimin Wang, Murali Ganesan, Franchesca Fonseca-Lanza, Denise A. Cobb, Grace Bybee, Yimin Sun, Lili Guo, Brandon Hanson, Samuel M. Cohen, Howard E. Gendelman, Natalia A. Osna, Benson J. Edagwa, Larisa Y. Poluektova

**Affiliations:** ^1^ Department of Pharmacology and Experimental Neuroscience, University of Nebraska Medical Center, Omaha, NE 68198, USA.; ^2^ Department of Pathology and Microbiology, University of Nebraska Medical Center, Omaha, NE 68198, USA.; ^3^ Department of Internal Medicine, University of Nebraska Medical Center, Omaha, NE 68105, USA.; ^4^ Research Service, Veterans Affairs Nebraska-Western Iowa Health Care System, Omaha, NE 68105, USA.

## Abstract

Treatment of chronic hepatitis B virus (HBV) requires lifelong daily therapy. However, suboptimal adherence to the existing daily therapy has led to the need for ultralong-acting antivirals. A lipophilic and hydrophobic ProTide was made by replacing the alanyl isopropyl ester present in tenofovir alafenamide (TAF) with a docosyl phenyl alanyl ester, now referred to as M1TFV. NM1TFV and nanoformulated TAF (NTAF) nanocrystals were formulated by high-pressure homogenization. A single intramuscular injection of NM1TFV, but not NTAF, delivered at a dose of TFV equivalents (168 milligrams per kilogram) demonstrated monthslong antiviral activities in both HBV-transgenic and human hepatocyte transplanted TK-NOG mice. The suppression of HBV DNA in blood was maintained for 3 months. Laboratory experiments on HBV-transfected HepG2.2.15 cells affirmed the animal results and the critical role of docosanol in the sustained NM1TFV antiviral responses. These results provide clear “proof of concept” toward an emerging therapeutic paradigm for the treatment and prevention of HBV infection.

## Teaser

A single dose of an ultra-long acting tenofovir prodrug suppresses HBV replication for at least three months in animal models.

## INTRODUCTION

The World Health Organization has recorded that more than 296 million people worldwide are infected with the hepatitis B virus (HBV). The infection causes more than 887,000 yearly deaths (*1*). Chronic hepatitis B (CHB) infection remains a substantial worldwide health care problem, with higher prevalence occurring in persons of Asian and Pacific Islander descent along with immigrants from sub–Saharan Africa. It also has substantial disease-associated morbidities and high mortality rates, especially in men who have sex with men, in people who use illicit drugs, and in people living with HIV-1 infection (*2*). Therefore, effective, long-acting therapeutics are urgently needed (*1*, *3*). Notably, adherence to antiviral therapy is required to suppress HBV replication and prevent the emergence of drug-resistant mutations. Suboptimal HBV virologic suppression can lead to end-stage liver disease (*4*). With this in mind, the need for potent, ultralong-acting (ULA) anti-HBV therapies cannot be overstated (*5*). Given its high potency and safety profile, tenofovir (TFV) is among the recommended HBV treatments. TFV-based prodrug regimens [TFV disoproxil fumarate/TFV alafenamide fumarate (TDF/TAF)] have also substantially reduced organ liver disease (*6*). However, fatigue from lifelong daily pills and adverse drug reactions have contributed to suboptimal therapy adherence and the associated emergence of drug resistance (*7*). To improve adherence, investigational implants containing TAF and entecavir have been developed with variable degrees of success (*8*–*10*).

By creating a TFV lipophilic ULA prodrug with inherent stability in extracellular matrices, improved drug absorption and distribution were realized in hepatocytes. Currently, there are no injectable ULA nucleotide/nucleoside analog reverse transcriptase inhibitors (NRTIs) for CHB treatment. We developed a modified pronucleotide (ProTide) to improve TFV’s physicochemical properties and efficacy. The ULA-modified TFV ProTide uses unconventional lipophilic and hydrophobic phosphoramidate linkages (*11*, *12*). The alanyl and short chain amino acid esters used to produce ProTides were substituted for more hydrophobic and bulky phenylalanine bearing a lipophilic docosyl ester to produce M1TFV (*13*). The modifications facilitated intracellular delivery of TFV active metabolites yielding improved drug half-life and sustained efficacy.

Toxicological studies affirmed the safety and tolerability of the M1TFV nanoformulation (*14*). Scale-up production of NM1TFV solid drug nanoformulations by high-pressure homogenization or wet bead milling has also been established, as are sustained tissue drug depots of parallel antiviral formulations (*15*). Notably, a single intramuscular injection of prodrug formulations into rodents and rhesus macaques can achieve sustained therapeutic drug concentrations at tissue viral replication sites that include the liver (*15*, *16*). Prodrug lipophilicity and slow tissue conversion affect sustained plasma drug levels. The delivery approach enables constant drug concentrations precluding the spikes and troughs seen by daily oral medicines and, hence, minimizing untoward side effects. ULA formulations can also serve to improve patient compliance, as was observed for people living with HIV (*17*). Similarly, maternal-fetal transmission would be reduced by ULA formulations (*18*), along with transmission and reactivation for patients on immunosuppressive therapy (*19*).

With these benefits in mind, we evaluated NM1TFV and compared its efficacy profiles against a nanoformulated TAF (NTAF) control using preclinical models of HBV in virus-infected mice with humanized liver and in HBV-transgenic animals. While intramuscularly administered NTAF was ineffective, the NM1TFV formulation suppressed HBV replication for 3 months after a single parenteral injection, suggesting enhanced and sustained liver drug delivery for the NM1TFV formulation.

## RESULTS

### NM1TFV sustains suppression of HBV DNA in transgenic mice

**Fig. 1.**
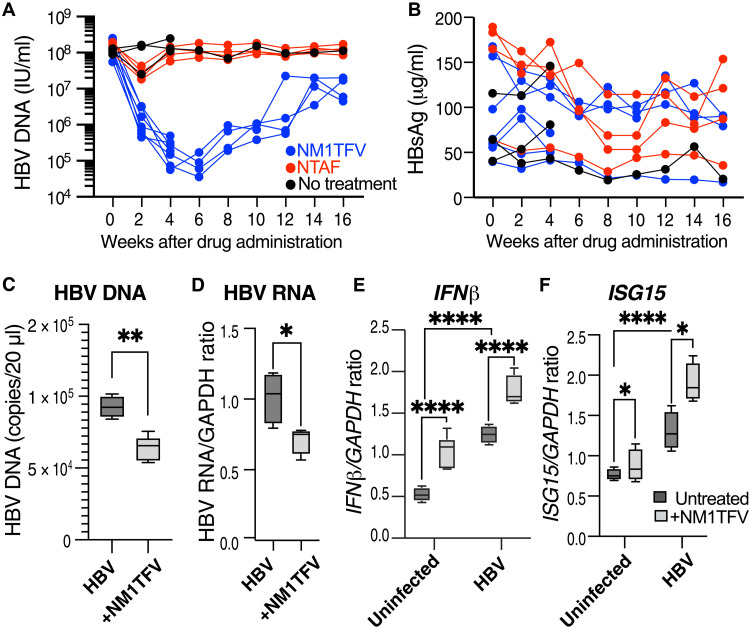
NM1TFV suppressed HBV peripheral blood viral load without affecting HBsAg concentration in transgenic mice and stimulated innate immune response. HBV-transgenic mice were administered a single intramuscular dose of NM1TFV (blue) or NTAF (red) at TFV equivalent (168 mg/kg; *n* = 4 per group and one left untreated; black dots). (**A**) NM1TFV suppressed HBV DNA in peripheral blood by 6 weeks with a slow rebound starting 8 weeks after injection. Individual mouse parameters are shown (A and B). Statistical analysis of differences between NM1TFV and NTAF results is shown in Table 1. (**B**) HBsAg concentration in peripheral blood showed minimal time-dependent decline. (**C** and **D**) Two untreated and three NM1TFV-treated HBV-transgenic mice labeled as HBV and +NM1TFV were euthanized at 4 weeks after drug administration for liver tissue analysis. NM1TFV reduced the expression of HBV DNA (C) and RNA in liver tissue (D). Three nontransgenic mice were untreated or NM1TFV-injected and euthanized at 4 weeks after drug administration (**E** and **F**). Transgenic expression of HBV up-regulated expression of *IFN*β (E) and *ISG15* (F). NM1TFV stimulated the expression of *IFN*β in liver tissue in nontransgenic and HBV-transgenic mice (E). *ISG15* expression was also up-regulated more significantly in HBV-transgenic mice (F). All data were analyzed in duplicate. Mean and SEM are shown. Statistical significance was calculated by the Mann-Whitney test (C and D) and two-way analysis of variance (ANOVA) (E and F) using GraphPad Prism 9, and only significant changes are indicated: *P < 0.05, **P < 0.01, and ****P < 0.0001.

HBV transgenic mice have a complete viral genome integrated into the chromosomes, which is constitutively transcribed at high levels in hepatic and renal cells (*20*). A single treatment with NM1TFV showed sustained suppression of HBV DNA in peripheral blood (Fig. 1 and Table 1). All animals had similar HBV DNA levels before treatment (8.16 ± 0.30, 8.18 ± 0.15, and 8.12 log_10_, respectively). During the treatment period, peripheral blood was assayed every 2 weeks. Analyses showed that NM1TFV suppressed viral load up to 2 log_10_ (*P* = 0.006), while NTAF had minimal effects (0.76 log_10_ reductions, *P* = 0.009) at 2 weeks after treatment. Notably, NM1TFV exhibited sustained suppression of HBV replication, and by week 6, the viral load dropped to 4.88 ± 0.29 (−3.28 log_10_). By contrast, NTAF showed no effect, and in this group, the HBV DNA concentration was equivalent to that of the untreated animals (Fig. 1A). During 8 to 12 weeks after NM1TFV injection, HBV DNA declined by ~2 log_10_ from the initial levels and rebounded by 16 weeks (Table 1). The levels of HBV surface antigen (HBsAg) in transgenic mice ranged from 50 to 200 mg/ml and showed a trend of time-dependent decline. At the early time points, there were no significant treatment-related changes (Fig. 1B). Furthermore, three HBV-transgenic mice treated with NM1TFV and two untreated animals were euthanized 4 weeks after treatment when a significant reduction in HBV DNA concentration in peripheral blood was observed (Fig. 1C). We also administered nontransgenic mice with an equivalent dose of NM1TFV. This was done to examine its effects on naïve mice. Murine liver tissue was collected, and the expression of HBV RNA, type I interferon-β (*IFNβ*), and IFN-stimulated gene (*ISG15*) were evaluated (Fig. 1, D to F). We found an increased expression of *IFN*β and *ISG15* RNAs in HBV-transgenic compared to nontransgenic mice (*P* < 0.0001). A single treatment with NM1TFV reduced the expression of HBV DNA and RNA in liver tissue (Fig. 1, C and D). *IFNβ* and *ISG15* expression were increased in the livers of HBV-transgenic mice (Fig. 1E) and induced further by NM1TFV (Fig. 1F).

**Table 1.** Analysis of treatment effects in HBV-transgenic mice.

Weeks after drug administration	HBV DNA log_10_^†^		Viral load reduction by log_10_ compared to week 0		Statistical significance of drugs effects, adjusted *P* values^††^	
	NM1TFV	NTAF	NM1TFV	NTAF	NM1TFV	NTAF
**0**	8.23	8.2				
**2**	6.28	7.44	−1.95	−0.76	0.006**	0.009**
**4**	5.14	8.03	−3.09	−0.17	0.005**	0.221
**6**	4.95	8.07	−3.28	−0.12	0.002**	0.477
**8**	5.71	8.01	−2.52	−0.18	0.004**	0.32
**10**	5.91	8.09	−2.32	−0.11	0.009**	0.684
**12**	6.81	7.99	−1.42	−0.21	0.053	0.28
**14**	7.14	8.09	−1.09	−0.11	0.000***	0.421
**16**	7.08	8.1	−1.15	−0.10	0.066	0.574

†Mean values are shown

††Two-way ANOVA analysis and Dunnett’s multiple-comparison *P* values of nontreated, NM1TFV-treated, and NTAF-treated mice;

**P < 0.01 and

***P < 0.001.

NM1TFV did not affect mouse body weight, and examination of tissue morphology showed no evidence of toxicity (fig. S1). In addition, staining for HBV core antigen (HBcAg) and HBsAg showed no significant differences in viral proteins expression in the liver (fig. S2). We analyzed the local muscle depot morphology at 4 and 16 weeks after injection (fig. S3). Infiltrated macrophages formed an early 4-week drug depot in NM1TFV-treated animals. However, by 16 weeks after NM1TFV injection, the local depot was involuted. Here, a more limited macrophage infiltrate was found surrounded by collagen. No evidence of muscle inflammation was observed.

### Docosanol and NM1TFV affect HBV replication and innate immune responses

**Fig. 2.**
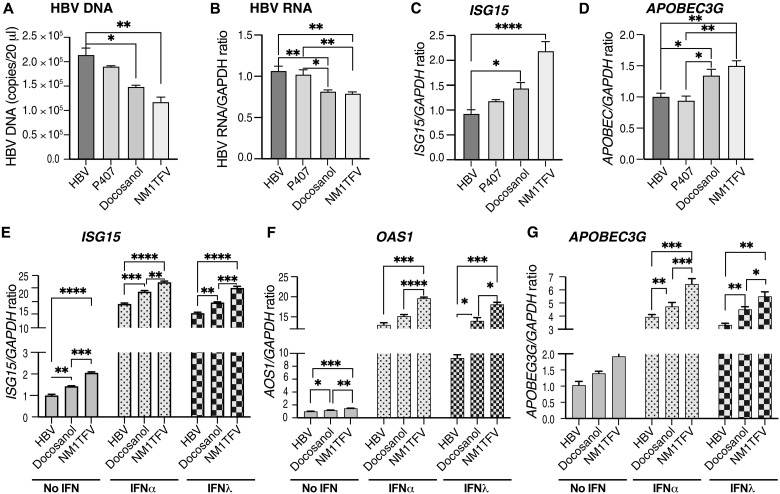
Docosanol and NM1TFV suppress HBV replication and stimulate an antiviral response to IFNs in vitro. In vivo results were supported by in vitro treatment of HBV-transduced HepG2.2.15 cells with 100 mM NM1TFV, P407-formulated docosanol, and P407 alone (**A** to **D**). Cells were exposed to compounds for 8 hours, washed, and cultured for 3 days. Docosanol and NM1TFV suppressed HBV DNA (A) and RNA (B) and up-regulated the expression of *APOBEC3G* and *ISG15* (C and D). To assess the effects of docosanol and NM1TFV on IFNs, the exogenous IFNα2 (400 IU/ml) and IFNλ (50 ng/ml) were added to the cells for the last 6 hours of incubation (**E** to **G**). The addition of IFNs to HepG2.2.15 cells significantly up-regulated the expression of *ISG15*, *OAS1*, and *APOBEC3G*. Docosanol and NM1TFV further increased the expression of these genes. In vitro experiments were done in quadruplicates, and mean and SEM are shown. Statistical significance was calculated by ordinary one-way ANOVA with Tukey’s multiple comparisons tests (A to D) and two-way ANOVA (E to G) using GraphPad Prism 9, and only significant changes are indicated: *P < 0.05, **P < 0.01, ***P < 0.001, and ****P < 0.0001.

To evaluate the contribution of docosanol to the antiviral activity of NM1TFV, we exposed HBV-transfected HepG2.2.15 cells to 100 μM NM1TFV, Pluronic 407 (P407)–stabilized docosanol formulation, or plain P407 (Fig. 2). After 8 hours of exposure, treatments were washed out, and cells were maintained in formulation free medium for 3 days. Docosanol exhibited a lower reduction in HBV DNA levels when compared to NM1TFV. The suppression of HBV RNA expression was similar in both docosanol- and NM1TFV-treated cells (Fig. 2, A and B). Docosanol increased IFN-stimulated gene 15 (*ISG15*) and apolipoprotein B mRNA editing enzyme catalytic subunit 3G (*APOBEC3G)* expression (Fig. 2, C and D). The up-regulation of ISGs could be due to increased production of IFN types 1 and 3 in both infected hepatocytes and neighboring immune cells sensing HBV infection as the result of innate immune responses. To evaluate whether the antiviral effects of docosanol and NM1TFV are at least partially related to ISG activation, we exposed untreated and drug-treated HepG2.2.15 cells to recombinant IFNs to measure the induction of antiviral genes, *ISG15*, 2′-5′-oligoadenylate synthetase 1 (*OAS1*), and *APOBEC3G*. As expected, IFNs activated antiviral genes in HepG2.2.15 cells with further increases made by docosanol and NM1TFV (Fig. 2, E to G).

### Plasma and tissue prodrug concentrations in HBV-transgenic mice sustained over 16 weeks

**Fig. 3.**
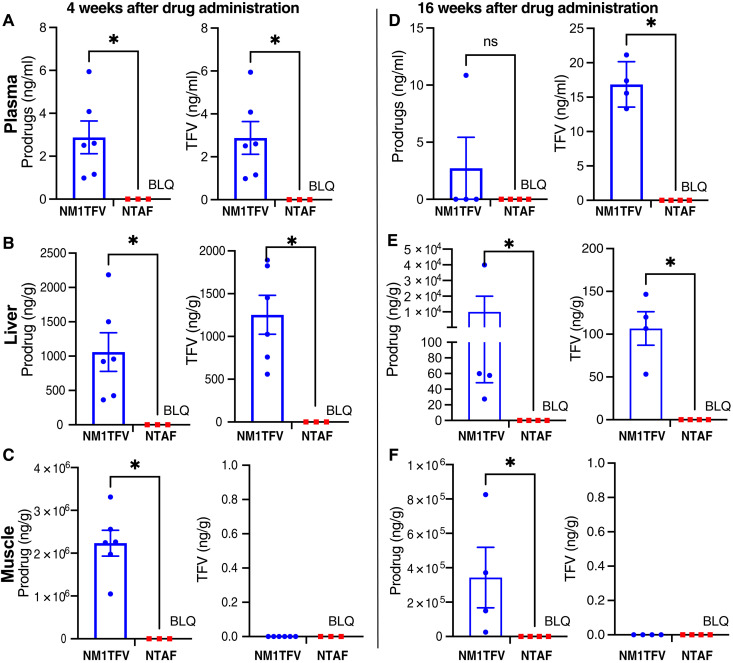
Plasma and tissue drug levels. HBV-transgenic mice were administered a single intramuscular dose of NM1TFV (blue) or NTAF (red) at TFV equivalent (168 mg/kg). (**A** to **F**) Each graph panel represents prodrug (left) and TFV (right) concentrations. Tissue biodistribution of M1TFV, TAF, and TFV was assessed in plasma (A and D), liver (B and E), and injection site (C and F) at 4 and 16 weeks after treatment. Data are expressed as means ± SEM, and dots represent individual mice. The limits of quantification (LQs) for M1TFV and TFV in plasma, liver, and muscle injection site were 186.7 and 41 pg/ml; 100 and 400 pg/ml; and 4.8 and 154.4 pg/g, respectively. BLQ, below levels of quantification. Prodrugs and TFV concentrations were compared using the unpaired *t* test with Welch’s correction. *P < 0.05 for NM1TFV compared to NTAF.

Treatment with NM1TFV produced sustained drug concentrations in the liver. Drug and prodrug levels at 4 and 16 weeks are shown in Fig. 3. At 4 weeks, the muscle depot contained substantial M1TFV levels (2.2 ± 0.3 mg/g). This declined to 0.34 ± 0.18 mg/g at 16 weeks. Drug and prodrug concentration in the liver was 1.3 ± 0.23 mg/g and 0.10 ± 0.02 mg/g and 1.06 ± 0.28 mg/g and 48.3 ± 10.4 ng/g at 4 and 16 weeks, respectively. NTAF-treated animals did not show detectable drug levels at either time point (Fig. 3).

### NM1TFV suppressed HBV for 3 months in the livers of humanized mice

**Fig. 4.**
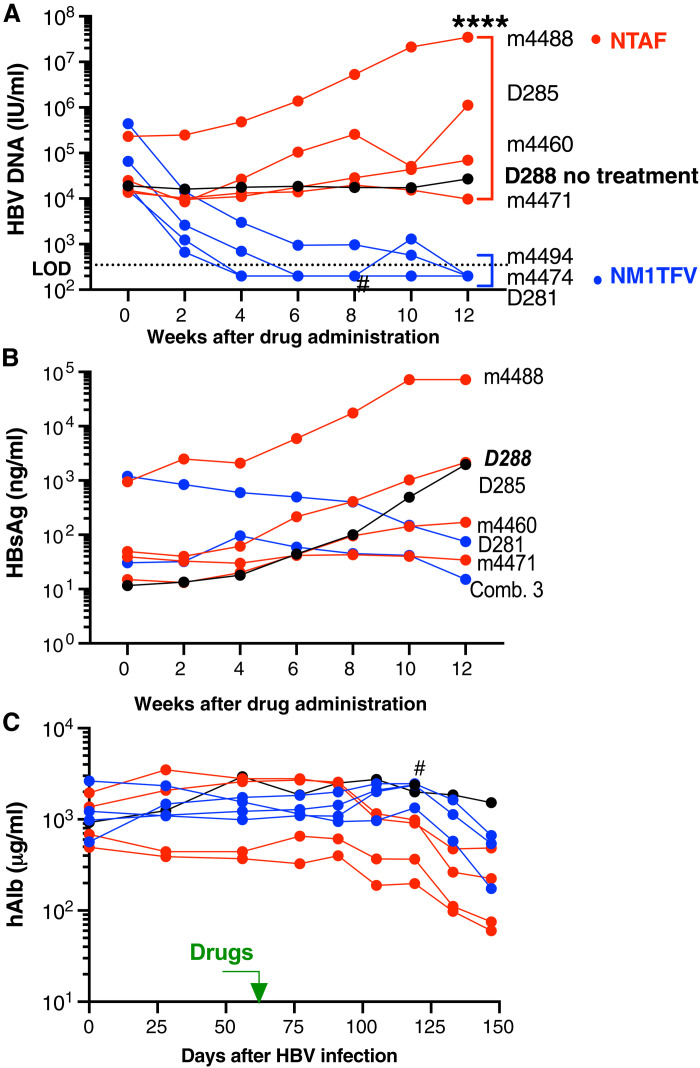
Suppression of HBV replication in humanized TK-NOG mice. Human hepatocyte transplanted and HBV-infected TK-NOG mice were administered a single dose of NM1TFV (blue) or NTAF (red) at TFV equivalent (168 mg/kg). (**A**) The dynamics of HBV DNA viral load in peripheral blood. NM1TFV suppressed viral replication below the limit of detection (LOD; 350 UI/ml) over 3 months. (**B**) The HBsAg concentration remains the same in combined samples from three animals or slightly declined in one mouse treated with NM1TFV but continues to grow in mice treated with NTAF or untreated transgenic control D288 (black symbols). (**C**) The levels of hAlb in peripheral blood. Control of HBV replication was not related to the partial loss of human hepatocytes associated with mice age. NTAF was not able to control HBV replication. #, lost mouse but not related to treatment. ****P < 0.0001 by one-way ANOVA between effects of NM1TFV and NTAF.

To evaluate the anti-HBV activity of NM1TFV, we transplanted humanized TK-NOG mice with human hepatocytes. After 2 months and following confirmation of human albumin (hAlb) in peripheral blood, the animals were infected with HBV intravenously with 10^6^ HBV DNA international units (IU) (*n* = 8). Following detection of HBV DNA in peripheral blood (week 0), a single intramuscular dose of TFV equivalent (168 mg/kg) of NM1TFV or NTAF was administered (Fig. 4). In animals treated with NM1TFV, HBV DNA levels in peripheral blood were reduced by 1.36 (*P* = 0.022), 2.00 (*P* = 0.004), and 2.26 (*P* = 0.001) log_10_ at 2, 4, and 6 weeks, respectively. HBV DNA levels remained below detection levels (350 IU/ml) in three of the four mice for up to 12 weeks (Fig. 4A). In the NTAF-treated group, HBV DNA levels remained stable or increased from baseline levels recorded in infected control mice. During the treatment period, HBsAg levels were tested by enzyme-linked immunosorbent assay (ELISA). HBsAg levels in the plasma of NTAF-treated or control (untreated mouse D288) mice increased over time. Notably, NM1TFV-treated mice showed a decline in blood HBsAg.

In humanized liver mice, HBV levels paralleled human hepatocytes engraftment as reflected by hAlb in blood. The levels of hAlb remained stable after HBV infection (Fig. 4C) up to 8 weeks after drug administration. The subsequent decline in hAlb reflects decreased human hepatocyte numbers corresponding to the animal’s age and the transplanted human cryopreserved cell integrity. However, an even more pronounced decline of hAlb was noted in NTAF-treated animals.

**Fig. 5.**
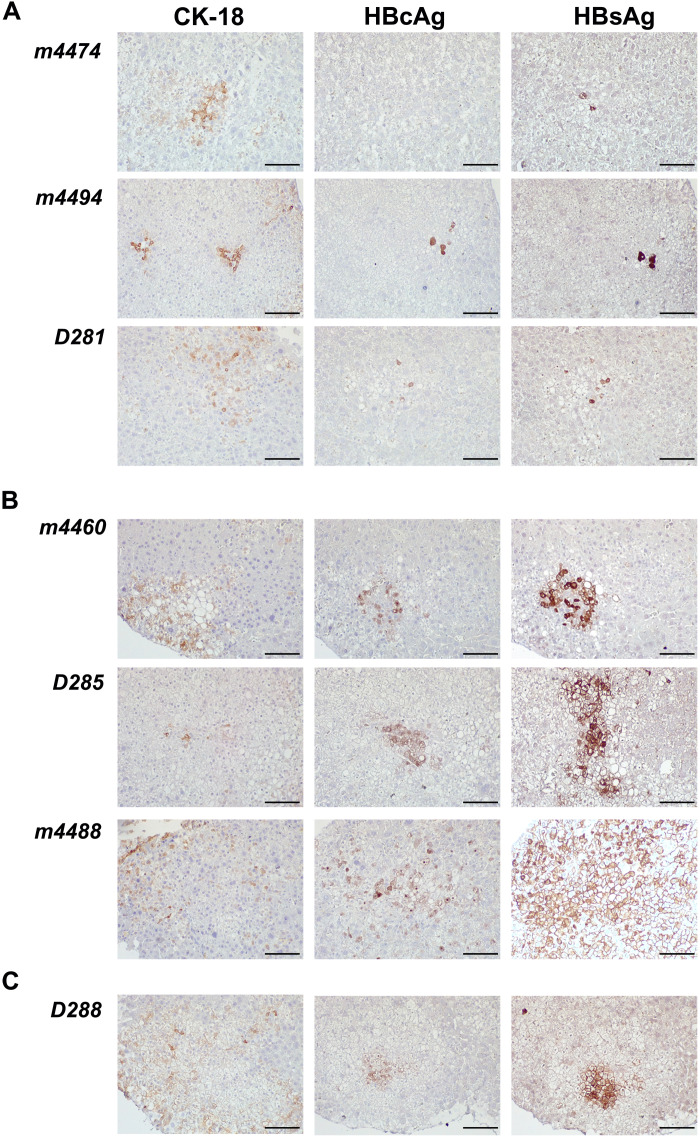
Immunohistological evaluation of chimeric liver samples from treated mice 3 months following drug administration. Liver humanized HBV-infected mice were administered a single dose of NM1TFV or NTAF at TFV equivalent (168 mg/kg). Animals were euthanized 12 weeks later. The liver tissue samples were fixed and embedded in paraffin, and 5-μm serial sections were stained for human CK-18 to identify areas of human hepatocytes, HBcAg, and HBsAg to confirm the presence of ongoing viral replication. The positive signal was visualized with 3'3-diaminobensidine (DAB) (brown). Sections were counterstained with hematoxylin. (**A**) Liver tissue samples for NM1TFV-treated mice contained a small number of viral protein–positive cells and did not have detectable levels of plasma virus (<350 UI/ml). (**B**) NTAF-treated mice retained a significant number of viral protein–positive cells that corresponded to the peripheral viral load. (**C**) Untreated animal D288 contained readily detectable infected cells. All images were captured under an original magnification of ×100. Scale bars, 100 μm.

Liver tissue sections collected at 12 weeks after drug administration were stained for human cytokeratin-18 (CK-18), HBcAg, and HBsAg (Fig. 5). Consistent with HBV DNA suppression in serum, HBcAg- and HBsAg-positive cells were reduced in NM1TFV-administered animals when compared to NTAF or untreated mice.

We also evaluated the expression of HBV RNA and HBV DNA in liver tissue samples by real-time polymerase chain reaction (RT-PCR) and digital droplet PCR (ddPCR), respectively (fig. S4). HBV DNA was detected in two of the three NM1TFV-treated mice (2620 and 29,200 copies/20 ml) and three of the four NTAF-treated mice (1180, 84,000, and 164,000 copies/20 ml). Human glyceraldehyde-3-phosphate dehydrogenase (GAPDH; human gene control) expression was detected in all animals, which confirmed the presence of human hepatocytes (fig. S4).

**Fig. 6.**
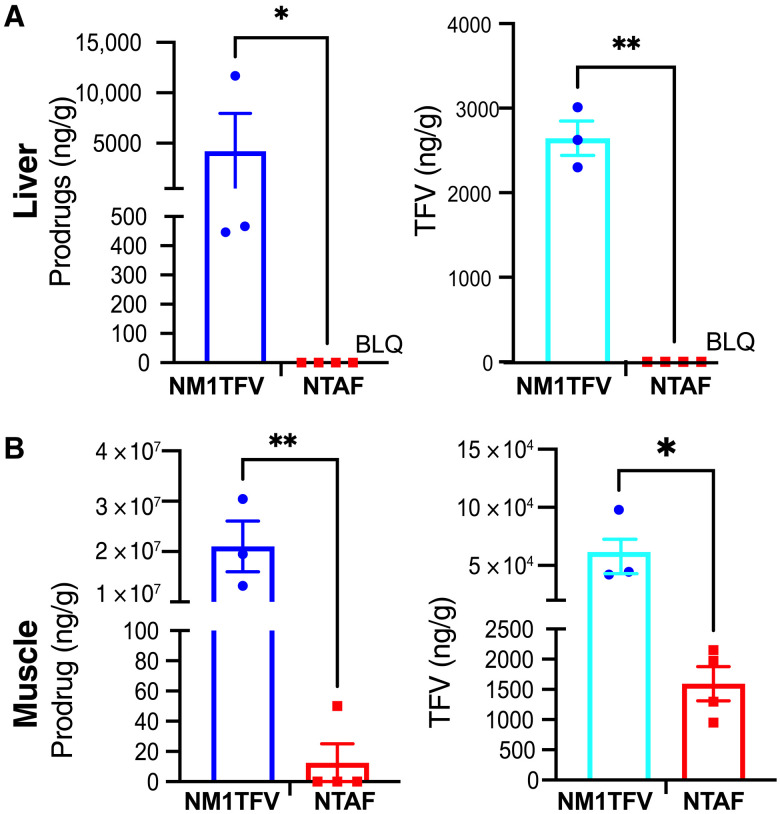
The concentration of TFV drug and prodrugs in tissues. (**A**) M1TFV prodrug was detectable in liver tissue, while TAF concentration was below levels of quantification (BLQ = 0.05 ng/g; left). A significant amount of TFV was detected in NM1TFV-injected animals (right). (**B**) Muscle tissue showed significant levels of M1TFV, and only one mouse showed detectable TAF levels in muscles. TFV was present in all samples. Data are expressed as means ± SEM, and dots represent individual mice. The LQ for M1TFV and TFV in liver and muscle injection sites was 0.05 ng/ml. Prodrugs and TFV concentrations were compared using the unpaired *t* test with Welch’s correction. *P < 0.05 and **P < 0.01 for NM1TFV compared with NTAF.

To elucidate how NM1TFV sustains the suppression of HBV replication, we analyzed the prodrug and drug concentrations in the liver and muscle (Fig. 6). At 12 weeks after NM1TFV injection, liver samples from all three animals had a significant amount of M1TFV and TFV (Fig. 6A). However, NTAF-treated mice did not have detectable TAF or TFV levels. These data served to affirm drug-related antiviral efficacy. We also collected the murine muscle tissue at the injection site. A sustained local drug depot was formed for NM1TFV-injected mice (Fig. 6B) but not NTAF. A significant amount of prodrug and TFV was quantified in the muscles of NM1TFV-treated animals. For NTAF-treated animals, TAF prodrug was detected only in one mouse sample, while TFV was detectable in all four animals in this treatment group (Fig. 6B). The NM1TFV injection site showed the presence of the drug as amorphous material and substantial infiltration of mouse macrophages. The injection site was demarcated with collagen deposition and a small number of vessels as an indication of ongoing vascularization (fig. S5). We observed no foreign-body giant cell reaction as observed in immune-competent animals (*15*).

## DISCUSSION

Notable reductions in cirrhosis and hepatocellular carcinoma and the prevention of vertical transmission were associated with the U.S. Food and Drug Administration–approved TFV therapy for CHB (*21*). We posit that further improved treatment outcomes can be achieved using long-acting injectable formulations. As now reported, these injectables enable the suppression of HBV DNA for 3 months or longer. HBV-active nucleoside (nucleotide) analogs, such as TFV prodrugs, have had a marked effect in lowering the incidence of acute HBV infections in HIV-1–infected patients (*22*). Moreover, HBV-active antivirals protect against the development of CHB (*23*) and improve the survival of patients with CHB-related hepatocellular carcinoma (*24*). TAF was designed to have greater plasma stability than TDF, enabling more efficient hepatic delivery of the pharmacologically active metabolite (TFV-diphosphate) when orally administered. TAF has proven to be as effective as TDF, with limited renal and bone adverse reactions in patients with CHB (*25*). However, poor regimen adherence and the desire to meet patient treatment needs have led to an emerging interest in long-acting injectable/implantable drug forms (*26*). Devices for sustained delivery of TAF were developed and tested in rodents, dogs, and nonhuman primates for Simian-Human Immunodeficiency virus (SHIV) prevention (*8*, *27*–*29*). A phase 1/2 clinical trial protocol to assess the safety, acceptability, tolerability, and pharmacokinetics (PK) of a sustained release TAF subdermal implant for HIV prevention in women was generated (CAPRISA 018), and the study is under way (*30*). However, there is no published report on ULA TFV injectables or implants for the treatment of CHB. While TAF-containing implants could provide a sustained target dose for HIV-1 prevention, adverse inflammatory responses at the implant tissue interface were recorded in dogs and nonhuman primates (*29*, *31*). This is likely due to extracellular, ionizable breakdown products from an unstable TAF prodrug at the injection site depot or counterions used to produce TAF salts. Elsewhere, subcutaneous administration of entecavir was found to induce local necrosis attributed to the hydrophilic nature of the antiviral agent that limits the absorption of the drug from the injection site depot (*32*). These implants are yet to be evaluated for anti-HBV efficacy. Another long-acting TFV formulation approach worth highlighting is a four-drug (TFV, lopinavir, ritonavir, and lamivudine)–loaded lipid nanoparticle that exhibited sustained drug levels in both plasma and peripheral blood mononuclear cells for 5 weeks after subcutaneous administration to *Macaca nemestrina* (*33*). However, in vivo efficacy for the multi-HIV drug–loaded lipid nanoparticle is yet to be demonstrated. Other notable efforts toward long-acting TFV formulations include poly lactic-*co*-glycolic acid (PLGA) nanoparticles loaded with a two-drug regimen consisting of TAF and emtricitabine. The PLGA nanoparticles protected humanized mice from vaginal HIV-1 challenge for 2 weeks after subcutaneous dosing at 200 mg/kg (*34*).

Our modified ProTide approach produced a ULA TFV formulation that demonstrates sustained HBV suppression with limited injection site reactions. The current study was based, in measure, on prior studies of long-acting lamivudine in TK-NOG mice reconstituted with human HBV-infected hepatocytes (*11*, *12*). We sought to extend these studies by generating NM1TFV and, hence, realizing a ULA of a potent compound with a high genetic barrier to resistance (*15*). TFV monophenol was modified by adding a phenyl alanyl docosyl ester to produce M1TFV (*13*). We then tested the efficacy of NM1TFV and TAF at TFV equivalent dosage in both HBV-infected humanized mice and transgenic animals.

To affirm the sustained antiviral efficacy of NM1TFV against HBV replication first observed in humanized mice, HBV-transgenic animals (Tg05) were used for antiviral testing (*35*). These animals constitutively transcribe HBV at high levels in hepatic and renal cells. In contrast to prior observations where treatment of HBV-transgenic mice with TFV exalidex administered at 10 mg/kg per day for 16 days suppressed HBV DNA levels (*36*), we observed a significant reduction by 6 weeks (>3 log_10_). The HBV DNA suppression was observed in the plasma of transgenic mice administered a single dose of NM1TFV. The former studies had more limited effects, while NTAF in the current report exhibited a <1 log_10_ reduction in HBV DNA at 2 weeks after drug injection.

In addition to HBV DNA reductions, we found no reduction of HBsAg. Unexpectedly, we found unique abilities of NM1TFV to stimulate innate immunity genes (type I IFN and *ISG15*) expression in the liver of transgenic and parental mice (Fig. 1). Previously, TFV was shown to augment IFNλ levels in the blood. In the laboratory setting, the drug was shown to stimulate ISGs by epithelial cells (*37*). We hypothesized that in addition to direct antiviral properties, NM1TFV has immunomodulating effects based on its ability to stimulate innate immunity. Here, we demonstrate that exposure of HBV-transduced hepatoma cell line HepG2.2.15 to docosanol alone suppresses HBV DNA and HBV RNA. These properties of docosanol could be related to the up-regulation of antiviral ISGs, such as APOBEC3G, which can edit up to 35% of the HBV genome (*38*). These antiviral activities were demonstrated at the early stages of viral reverse transcription (*39*).

While separate antiviral effect studies on the induction of IFN-stimulated genes, *ISG15*, *OAS1*, and *APOBEC3G* by docosanol were variable, NM1TFV stably up-regulated their expression. We cannot exclude a possible increase in IFN type 1 and type 3 production by HepG2.2.15 cells triggered by treatment alone and due to suppression of HBV infection. However, the activation of antiviral genes in infected hepatocytes is regulated via the Janus kinase–signal transducers and activators of transcriptions 1 and 2 pathways, not only because of sensing of intrinsic IFN but also because of IFNs released by adjacent immune cells. This sensing requires receptors expressed on the infected hepatocyte surface to initiate the signaling. Consequently, in HepG2.2.15 cells, we observed the induction of *ISG15*, *OAS1*, and *APOBEC3G* mRNAs by both IFNs, which was further potentiated by NM1TFV, suggesting that in addition to direct antiviral properties, NM1TFV promotes antiviral activity by triggering protective innate immunity.

Each of the antiviral observations made in the current report was affirmed in divergent model systems. This included transgenic mice, HBV-transduced cells, and a humanized liver mouse model. In humanized liver TK-NOG mice, a single intramuscular injection of NM1TFV significantly suppressed HBV DNA in peripheral blood, declining it below the detection level by 12 weeks after injection. By contrast, NTAF exhibited minimal effect, suggesting inefficient drug delivery into the liver when the route of TAF administration is changed from oral to parenteral. In addition, a higher dose of TAF will likely be needed to suppress viral replication. The efficacy of NM1TFV significantly exceeds that of other reported TFV prodrugs in humanized mouse models (*40*, *41*). Using mice transplanted with HepAD38 hepatoma cells, others demonstrated that the administration of TDF at 300 mg/kg for 6 days (intraperitoneally) maintained HBV suppression at 23% for up to 10 days after treatment cessation (*41*). In a separate study, a novel 4′-modified NRTI, (1S,3 S,5 S,E)-3-(2-amino-6-oxo-1,6-dihydro-9*H*-purin-9-yl)-2-(fluoromethylene)-5-hydroxy-1-(hydroxy-methyl)cyclopentane-1-carbonitrile potently blocked the production of wild-type and drug-resistant HBV in chimeric mouse livers. These observations were made after oral administration (0.2 mg/kg) for 2 weeks (2.6 log_10_ suppression) with sustained effects for 3 weeks after treatment cessation (*42*). Notably, NM1TFV suppressed HBV replication over 3 months. Sustained prodrug concentration in liver tissues and injection sites was observed (Fig. 6). The prolonged action of NM1TFV was related to the physicochemical properties of the prodrug suspension and the formation of drug depots at the injection site and liver.

In conclusion, a single intramuscular injection of NM1TFV strongly suppresses HBV replication for up to 3 months. This ULA effect derives in part from antiviral innate immunity triggered by NM1TFV. This prodrug formulation could potentially improve adherence and limit viral transmission. Reductions in seroconversion and HBsAg would facilitate finite treatment options for CHB infections. We posit that multiple groups of patients will benefit from ULA TFV that include, but are not limited to, CHB and persons coinfected with HIV-1 or with complex clinical comorbidities. This formulation could also facilitate HIV-1 and HBV viral prophylaxis.

## MATERIALS AND METHODS

### Preparation of the nanoformulations

The synthesis and characterization of TFV ProTide were previously described (*15*). NM1TFV and NTAF solid drug nanoparticles were manufactured by high-pressure homogenization in aqueous buffers stabilized by nonionic surfactants. We used a 2:1 (w/v) prodrug to surfactant ratio, while the suspension concentration was in the range of 0.1 to 15% (w/v) for the drug/prodrug and 0.05 to 8% (w/v) for P407. NM1TFV nanoformulation was prepared in P407 surfactant solution in 10 mM Hepes at pH 7. For NTAF, a mixture of P407 and polyethylene glycol 3350 (PEG-3350) was used as stabilizers. Attempts to formulate TAF using P407 without PEG-3350 produced unstable formulations. Specifically, TAF was dispersed in a P407/PEG-3350 solution in 10 mM Hepes (pH 5.5) and allowed to form a presuspension. The prodrug to P407-to-PEG-3350 ratio was maintained at 4:1:1 (w/v), and the suspension concentration was in the range of 0.1 to 15% (w/v) for the drug/prodrug and 0.025 to 4% (w/v) for P407/PEG. The presuspensions were homogenized on an Avestin EmulsiFlex-C3 high-pressure homogenizer at 15,000 to 20,000 psi until the desired particle size of 200 to 500 nm was achieved. The homogenized solid drug nanoparticles were then evaluated for particle size, homogeneity, and surface charge by dynamic light scattering using a Malvern Zetasizer Nano-ZS. Long-term nanoparticle stability for all formulations during storage was evaluated at room temperature over a period of 3 months. After homogenization, the amount of TAF and M1TFV within the formulations was determined from diluted formulation samples in MeOH (1000- to 10,000-fold dilution) and analyzed by Ultra Perfomance Liquid Chromotography (UPLC)–ultraviolet-visible spectroscopy using calibration curves with known standards. The percentage of drug entrapped within each nanosuspension was calculated using the equation: encapsulation efficiency (%) = (weight of the drug in formulation/initial weight of drug added) × 100.

### HBV-transgenic mice

The study was approved by the Institutional Animal Care and Use Committee of the University of Nebraska Medical Center (#19-034-10FC). The Animal Research: Reporting of In Vivo Experiments guidelines were used to prepare the data assembled in the current manuscript. Animals were housed under pathogen-free conditions and received a standard diet. C57BL/6 male transgenic Tg05 mice expressing 1.3 HBV of wild-type viral genome were obtained from J.-H. James Ou, University of Southern California (*35*). Mice were maintained in a pathogen-free barrier facility.

### Humanized liver TK-NOG mouse model

NOD.Cg-*Prkdc^scid^ Il2rg^tm1Sug^* Tg(Alb-UL23)7-2/ShiJic (TK-NOG) mice are a specially designed strain (*43*) that allows for the conditional depletion of mouse hepatocytes and engraftment of their human cell counterparts. We used these animals as previously described (*12*). Eight- to 10-week-old TK-NOG male mice were selected by genotyping and injected with ganciclovir (10 and 30 mg/kg) 7 and 5 days to achieve significant damage of mouse liver as evident by elevated to ALT levels (200 to 400 IU/ml) before transplantation of human hepatocytes. Human hepatocytes were obtained from Lonza (lot no. 4145; Walkersville, MD, USA). Two million hepatocytes were intrasplenically infused. The levels of hepatocyte engraftment were monitored starting from 1 month after transplantation. The chimerism rate correlated with serum hAlb levels (*43*) measured using the Human Albumin ELISA Kit (Bethyl Laboratories Inc., Montgomery, TX). At 2 months after transplantation, animals were intravenously infected with patient-derived sera samples containing ~10^6^ HBV DNA, and the drug treatment was started at 2 months after infection. Overall, the mice were followed for 3 months after drug injection. The duration of experimental animal life reached ~9 months.

### Animal treatment

On the basis of PK studies performed in Sprague-Dawley rats at a dose of 75 mg/kg (*15*), a dose of TFV equivalents (168 mg/kg) was selected for mice efficacy studies. For HBV-transgenic mice, four mice were injected with NTAF and four others with NM1TFV. There were equal numbers of males and females. One animal was left without treatment. Blood was collected biweekly for 16 weeks. In the second transgenic mouse experiment, two animals were used as controls, and three were administered NM1TFV. The animals in the second transgenic mouse experiment were euthanized 4 weeks after injection. Nontransgenic mice were used to study the effects of NM1TFV on antiviral gene expression in liver tissues. Next, we evaluated the formulations in humanized liver TK-NOG mice at 8 weeks after HBV infection and when viral DNA levels were readily detected in peripheral blood. Specifically, humanized HBV-infected mice were administered a single intramuscular 50-μl injection of NM1TFV or 30 μl of NTAF at a dose of TFV equivalent (168 mg/kg) in the caudal thigh muscle. Animals were evaluated for viral suppression biweekly for 3 months.

### Measurement of tissue drug levels

We measured the tissue concentration of TFV, TAF, and M1TFV by fitting UPLC–tandem mass spectrometry (MS/MS) data to standard calibration curves on a Waters ACQUITY H-class UPLC connected to a Xevo TQ-S micro–mass spectrometer. The solvents used for sample processing and analytical methods were all MS-grade (*15*).

### Nanoformulated treatments in HepG2.2.15 cells

HepG2.2.15 cells were cultured as previously described (*44*). Cells were exposed to 100 μM NM1TFV, vehicle control (P407), or P407 docosanol stabilized formulation for 8 hours and then washed and monitored for 3 days in formulation-free culture medium. DNA and RNA were then extracted, and the expression of HBV RNA and DNA, APOBEC3G, and ISG15 were evaluated.

### HBV DNA in blood

Starting at 1 month following viral infection, HBV DNA levels were measured in peripheral blood using the COBAS TaqMan HBV Test (Roche Diagnostics, Switzerland). The lower detection limit was 20 IU/ml (1 IU = 5.6 DNA copies). The samples were diluted 17.5-fold, and the detection limit was 350 IU/ml.

### Measurements of plasma HBsAg

Murine plasma was stored at −80°C and analyzed for HBsAg concentration by a sensitive (0.31 to 20 ng/ml) quantitative ELISA kit (Abbexaâ, Zoetermeer, NL) following the manufacturer’s instructions.

### HBV DNA and RNA and immune factor detection in liver tissue

HBV DNA copy number was quantified by ddPCR (*44*). HBV RNA and innate gene expression were monitored as previously described (*44*). The latter included *ISGs*, *OAS1*, *APOBEC3G*, and *ISG15* measured by RT-PCR using methods developed for HBV RNA (*45*). Reagents for RNA and DNA isolation, cDNA synthesis, and RT-PCR were from Life Technologies (Carlsbad, CA). Total RNA was isolated from cells using TRIzol reagent. A two-step procedure was applied, in which 200 ng of RNA was reverse-transcribed to cDNA using the High-Capacity Reverse Transcription Kit. Then, the cDNA was amplified using TaqMan Universal Master Mix-II with fluorescent-labeled primers (TaqMan gene expression systems). After incubation in a model 7500 quantitative RT-PCR thermal cycler, the relative quantity of each RNA transcript was calculated by its threshold cycle (*C*_t_) after subtracting that of the reference cDNA (GAPDH). Data were expressed as the quantity of transcript by relative quantification (RQ). HBV infection was confirmed by measuring HBV RNA (single vial -primer-probe). The relative HBV RNA expression levels in infected cells were quantified using the primers and probes from Applied Biosystems and Life Technologies by Thermo Fisher Scientific, CA, and the sequence used 5′-CGTCTGTGCCTTCTCATCTGC-3′, 5′- GCACAGCTTGGAGGCTTGAA-3′, and probe FAM-CTGTAGGCATAAATTGGT.

Total DNA was prepared using the DNeasy Kit (QIAGEN, Germany) according to the manufacturer’s protocol. The concentrations of DNA were quantified using the QX200 Droplet Digital PCR System (Bio-Rad, Hercules, CA) according to the manufacturer’s instructions. Briefly, we used 20 μl of ddPCR reaction comprised 2× ddPCR Supermix (5 μl), reverse transcriptase (2 μl), 300 mM dithiothreitol (1 μl; Bio-Rad, Pleasanton, CA), 900 nmol per HBV sense (5′-CGACGTGCAGAGGTGAAG-3′),and antisense (5′-CACCTCTCTTTACGCGGACT-3′) primers, 250 nmol of HBV probe (5′-/56-FAM/ATCTGCCGG/ZEN/ACCGTGTGCAC/3IABkFQ/-3′), and 5 μl of adjusted DNA sample in ribonuclease-free water. Primers and probes were from Integrated DNA Technologies (Coralville, IA). Prepared droplets were transferred to corresponding wells of a Bio-Rad 96-well PCR plate, using an Automated Droplet Generator as described in the instruction manual (no. 10043138). The PCR plate was subsequently heat-sealed with pierceable foil using the PX1 PCR plate sealer (Bio-Rad, Hercules, CA) and then amplified in the C1000 Touch deep-well thermal cycler (Bio-Rad). The ddPCR data were analyzed using QuantaSoft analysis software (Bio-Rad) (*44*).

### Immunohistochemistry

Tissues were fixed with 4% paraformaldehyde overnight at 4°C and then embedded in paraffin. Five-micrometer sections were cut from the paraffin blocks, mounted on glass slides, and subjected to immunohistochemical staining following procedures previously described (*12*). We used mouse monoclonal antibodies for CK-18 (clone DC10; 1:33 dilution) from Thermo Fisher Scientific, HBcAg (clone LF161; 1:50 dilution), and rabbit polyclonal antibodies to HBsAg (PAB361C; 1:50), both from Innovex Biosciences, Richmond, CA. Mouse monoclonal antibodies to α–smooth muscle actin were obtained from Abcam (clone 1A4; 1:500 dilution; Cambridge, United Kingdom). Polymer-based horseradish peroxidase–conjugated anti-mouse systems were used as secondary detection reagents and were developed with 3,3′-diaminobenzidine. All paraffin-embedded sections were counterstained with Mayer’s hematoxylin. Bright-field images were obtained with a Leica DM1000 light-emitting diode under an original magnification of ×100. The staining for connective tissue at the injection site was done with a Picro Sirius Red stain kit (Abcam, ab150681) according to the manufacturer’s instructions.

### Statistical analysis

Statistical significance was determined using GraphPad Prism version 9.4.1 by one-way and two-way analyses of variance, *t* tests, with the *P* ≤ 0.05 being considered significant. Experimental details are expanded within the figure legends.

## Notes

View/request a protocol for this paper from *Bio-protocol*.
