# Supplementary material for: An ultralong-acting tenofovir ProTide nanoformulation achieves monthslong HBV suppression
Source: Sci Adv. 2022 Dec 23;8(51):eade9582. doi: 10.1126/sciadv.ade9582 (PMC9788773; doi:10.1126/sciadv.ade9582)
Supplement: Supplementary file 1 — Figs. S1 to S5 [file sciadv.ade9582_sm.pdf]

Science Advances
AAAS

Supplementary Materials for

An ultralong-acting tenofovir ProTide nanoformulation achieves monthslong HBV suppression

Srijanee Das *et al.*

Corresponding author: lpoluekt@unmc.edu; benson.edagwa@unmc.edu; nosna@unmc.edu

Sci. Adv. **8**, eade9582 (2022)
DOI: 10.1126/sciadv.ade9582

This PDF file includes:

Figs. S1 to S5

Supplementary Figures

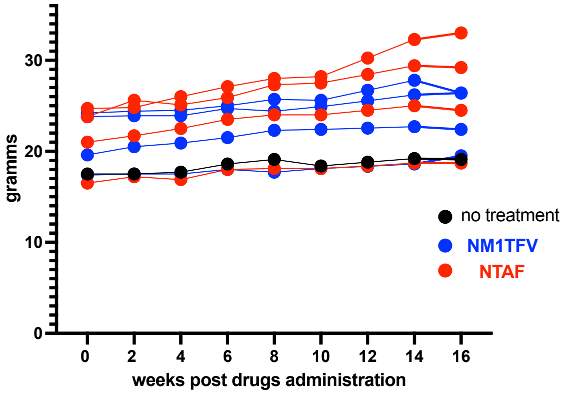

Fig. S1. HBV-transgenic mice body weight. HBV-transgenic mice administered with NM1TFV or NTAF retained stable body weight during observation.

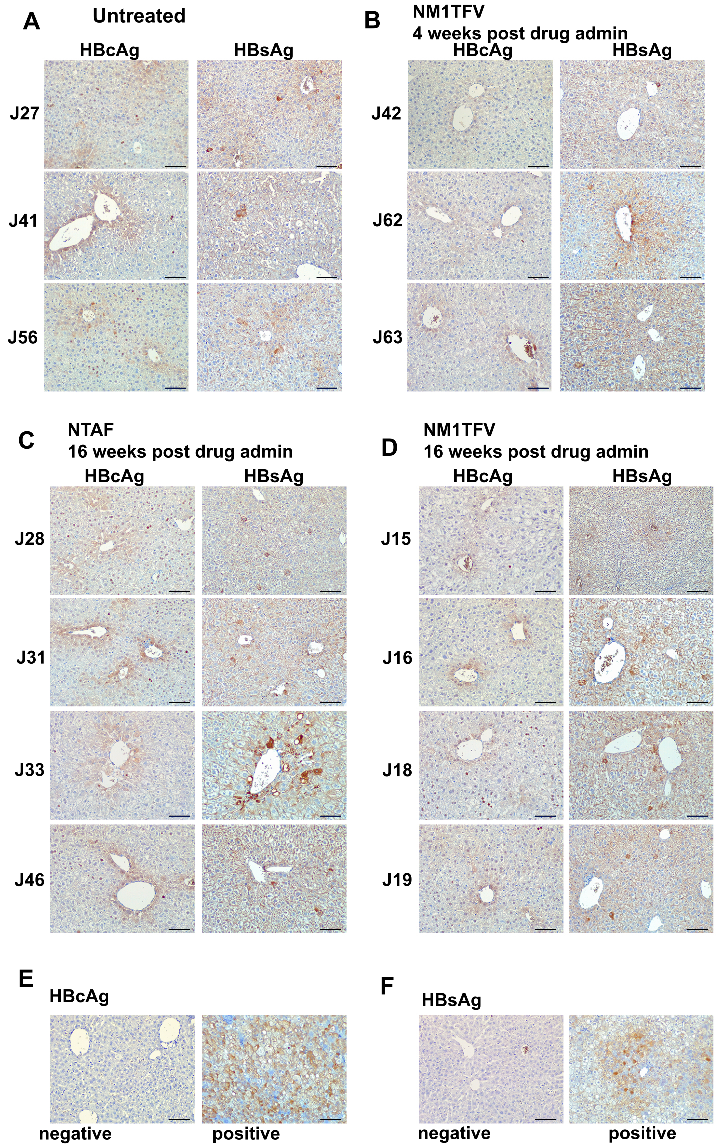

Fig. S2. Immunohistology of HBV transgenic mice liver tissues. Individual fixed and paraffin-embedded liver tissue samples from untreated (**A**), at four weeks post NM1TFV administration (**B**), and sixteen weeks post administration of NTAF (**C**) and NM1TFV(**D**) were stained for HBcAg and HBsAg. (**E** and **F**) Negative and positive control staining of human hepatocytes. All images were captured under an original magnification of 100×. Scale bars are 100 µm.

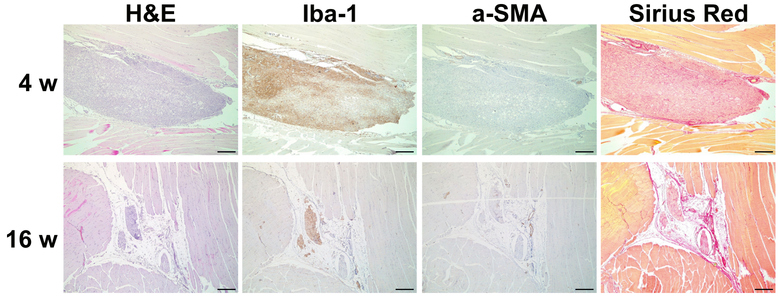

Fig. S3. Injection site evaluation for drug depot and foreign body reaction following intramuscular administration of NM1TFV nanocrystals. A primary injection site drug depot was formed for NM1TFV but not for NTAF. Injection site muscle samples were collected at four and sixteen weeks. One representative muscle sample from mice injected with NM1TFV is shown. Tissues were fixed and paraffin embedded. Five-micron thick sections were stained for the presence of infiltrating macrophages with hematoxylin and eosin (H&E) and antibodies for Iba-1. The drug depot was heavily infiltrated by macrophages at four weeks and almost resolved by sixteen weeks post-injection. The formation of small vessels was detected by alpha-smooth muscle actin (α-SMA) staining. Collagen deposition was visualized by Sirius Red staining. The drug depot was not surrounded by strong collagen deposition, and muscle remained intact. There was no evidence of muscle degeneration, basophils or eosinophils, cellulitis, or abscesses. All images were captured under an original magnification of 40×**.** Scale bars are 200 μm.

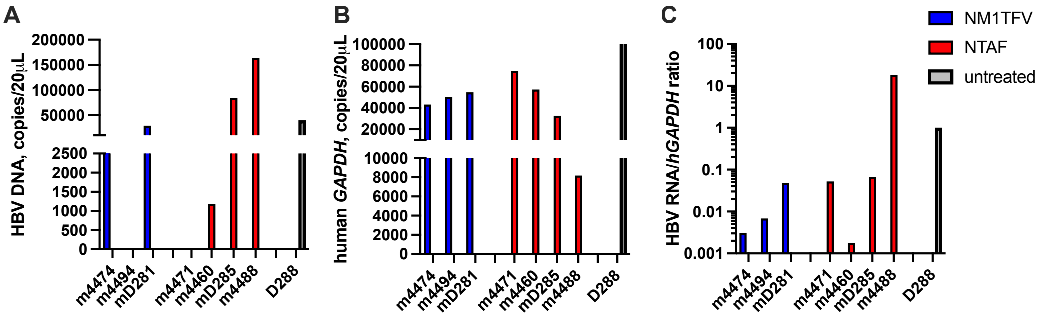

Fig. S4. Evaluation of HBV DNA and RNA in humanized liver samples. Randomly collected humanized liver tissue samples were analyzed for the presence of HBV DNA and RNA by ddPCR and RT-PCR, respectively. The levels of HBV DNA were higher in two of three NTAF-treated mice compared to detectable in two NM1TFV-treated animals (**A**). The detection of human GAPDH confirmed a relatively similar presence of human hepatocytes (**B**). The reduction of HBV RNA was found in two of the three analyzed mice compared to NTAF-treated and non-treated animals (**C**).

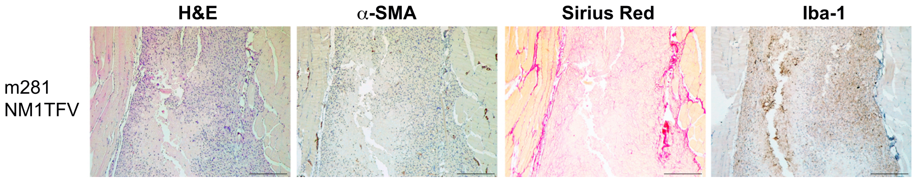

Fig. S5. Injection site evaluation for drug depot and foreign body reaction following intramuscular administration of NM1TFV nanocrystals. A primary injection site drug depot was formed for NM1TFV but not for NTAF. Injection site samples were stained for the presence of fibrotic demarcation of foreign material (Sirius Red) and infiltrating macrophages (Iba-1). There was no evidence of muscle degeneration, basophils or eosinophils, cellulitis, or abscesses. The formation of small vessels was detected by alpha-smooth muscle actin (α-SMA) staining. All images were captured under an original magnification of 100×. Scale bars are 100 μm.
